# Unveiling the Heart of the Matter: Intravascular Ultrasound in Acute Myocardial Infarction

**DOI:** 10.7759/cureus.47020

**Published:** 2023-10-14

**Authors:** Juan I Vazquez-Fuster, Victor H Molina-Lopez, Ismael Ortiz Cartagena

**Affiliations:** 1 Cardiology, Veterans Affairs (VA) Caribbean Healthcare Systems, San Juan, PRI; 2 Cardiology, Pavia Hospital, San Juan, PRI

**Keywords:** acute myocardial infarction, primary percutaneous coronary intervention (pci), interventional cardiologist, acs (acute coronary syndrome), substance induced disorders, left main coronary artery disease, acute st-elevation myocardial infarction, intravascular ultrasound (ivus)

## Abstract

Intravascular ultrasound (IVUS) has become crucial in contemporary percutaneous coronary interventions (PCIs), offering detailed two-dimensional (2D) arterial wall visualization. Current guidelines consider it valuable for guiding coronary stent placement, especially in complex cases like the left main (LM) artery, allowing a comprehensive assessment of vessel characteristics and stent performance. There are some studies that highlight the potential impact of IVUS on acute myocardial infarction (AMI) management, notably improving outcomes. This case involves a 37-year-old man who experienced an AMI, necessitating the use of IVUS to ascertain the underlying cause of his acute coronary syndrome (ACS). This approach was essential for guiding appropriate treatment and ultimately led to successful stent implantation.

## Introduction

Intravascular ultrasound (IVUS) has proven to be an essential tool in contemporary percutaneous coronary interventions (PCIs). Introduced by Yock et al. in the 1980s, these catheters use sound waves to visualize the arterial wall in a two-dimensional (2D), tomographic format [1]. It is an intravascular imaging modality primarily used in interventional cardiology to characterize lesion morphology, quantify plaque burden, guide stent sizing, assess stent expansion, and identify procedural complications [2]. With over 20 randomized controlled trials, over 30 meta-analyses, and over 80 registries, it is believed by many that it should be routinely incorporated into PCI procedures, especially in high-risk and complex lesions [3]. While studies have shown the benefit of IVUS in acute myocardial infarction (AMI), its routine use in this clinical scenario is still uncertain [4]. We present the case of a 37-year-old man who came to our institution due to an AMI who required IVUS to establish the etiology of his acute coronary syndrome (ACS), which led to successful stent implantation.

## Case presentation

**Figure 1:**
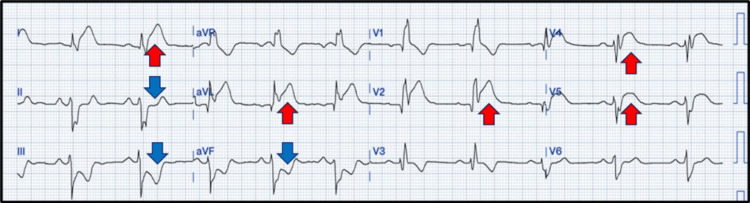
The ECG shows normal sinus rhythm with a right bundle branch block with anteroseptal and anterolateral ST-segment elevation (red arrows) and reciprocal ST depression in the inferior leads (blue arrows).

A 37-year-old man, previously diagnosed with polycythemia vera (PV) and a history of marijuana use, presented to the emergency department with a 45-minute history of left-sided, non-radiating, retrosternal chest pain that was somewhat alleviated by sublingual nitroglycerin provided upon arrival with associated diaphoresis. The patient admitted to non-compliance with antiplatelet therapy prescribed for his chronic hematological condition and disclosed marijuana use before arriving at the hospital. Upon arrival, his vital signs were borderline low at 96/62 mmHg. Physical examination did not reveal heart murmurs or indications of volume overload, although the patient was hypoactive and acutely unwell due to chest pain. The EKG was remarkable for a right bundle branch block with anteroseptal and anterolateral ST elevations and reciprocal ST depression in the inferior leads (Figure 1).

**Figure 2:**
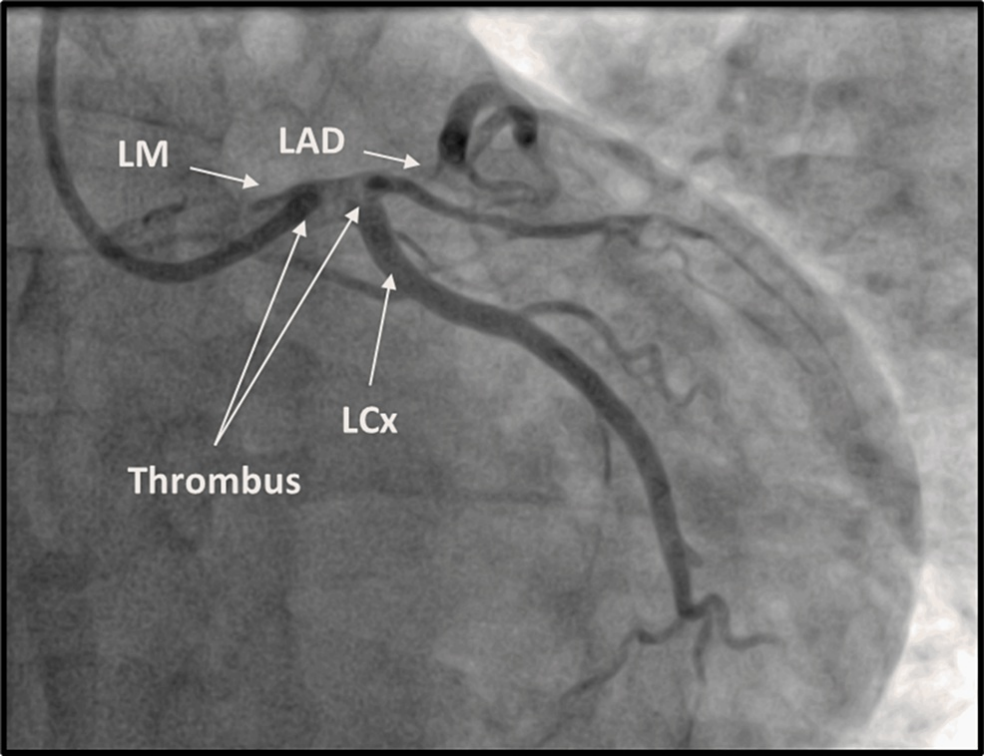
Anteroposterior caudal view showing extensive thrombus in the left main (LM) artery leading to an underfilled left anterior descending (LAD) artery with thrombolysis in myocardial infarction (TIMI)-1 flow. The left circumflex (LCx) showed no angiographic evidence of obstructive disease.

The clinical picture was convincing for ST-elevation myocardial infarction (STEMI), which was why he was taken for an emergent cardiac catheterization. Through radial access, the left main (LM) artery was engaged using a guide catheter, and the angiography showed a large filling defect consistent with a thrombotic lesion extending from the LM artery to the left anterior descending (LAD) artery with thrombolysis in myocardial infarction (TIMI) 1 flow (Figure 2). 

**Figure 3:**
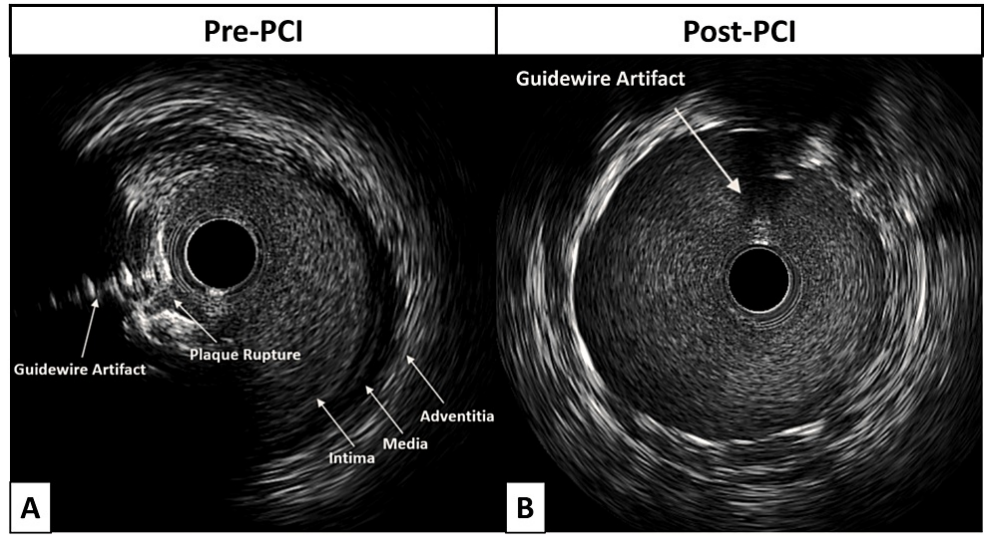
(A) Intravascular ultrasound (IVUS) of the proximal left anterior descending (LAD) artery shows a significant plaque burden, confirming plaque rupture as the cause of the patient's ST-elevation myocardial infarction (STEMI). (B) The IVUS post-percutaneous coronary intervention (PCI) shows adequate stent expansion and apposition, with no edge dissections.

The LAD artery was crossed using a coronary guidewire, and manual aspiration thrombectomy was performed, restoring TIMI-3 flow after two passes. Intracoronary nitroglycerin and nicardipine were provided at that point to improve distal flow. The IVUS imaging of the LM and LAD arteries confirmed a plaque rupture as the cause of the patient's STEMI, with a significant ostial LAD plaque burden (Figure 3A).

**Figure 4:**
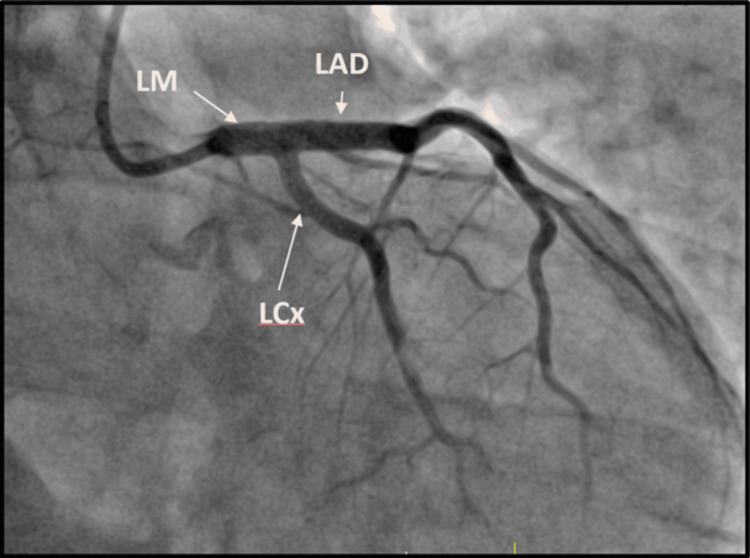
Right anterior oblique caudal view after percutaneous coronary intervention, showing an adequately expanded stent without dissections, distal embolization, or perforations.

After predilation with a 3.5x20mm balloon, a 4.5x32mm drug-eluting stent was deployed from the LM artery into the proximal LAD artery due to the challenging plaque burden in the ostial LAD, making it an unsuitable “landing zone” for stenting. Subsequent post-dilation of the stent was carried out using a 5.0x15 mm non-compliant balloon. The IVUS affirmed proper stent expansion and apposition, with no edge dissections and negligible plaque burden near the stent’s boundaries (Figure 3B). The decision not to perform balloon dilatation through the stent struts into the left circumflex was justified by the absence of angiographic disease in the ostium and the near-perpendicular angle from the LM artery. Final angiography with orthogonal views confirmed adequate stent expansion, TIMI-3 flow, and the absence of dissections, distal embolization, or perforations (Figure 4).

## Discussion

Percutaneous coronary interventions have revolutionized the management of coronary artery disease, offering effective means of restoring coronary blood flow. While coronary angiography remains the gold standard for guiding these procedures, IVUS has emerged as a valuable adjunctive tool [2]. Numerous studies and clinical trials have shed light on IVUS's role in AMI management, collectively underscoring its influence on procedural decision-making and post-PCI outcomes. The pivotal MATRIX (Comprehensive Assessment of Sirolimus-Eluting Stents in Complex Lesions) registry demonstrated significant reductions in both short- and long-term outcomes with IVUS compared to angiography alone [5]. The ULTIMATE (Intravascular Ultrasound-Guided Drug-Eluting Stents Implantation in "All-Comers" Coronary Lesions) trial showed improved clinical outcomes with IVUS-guided drug-eluting stent (DES) implantation, particularly for patients with an IVUS-defined optimal procedure [6]. Kim et al. examined the long-term impact of IVUS-guided PCI in AMI patients, revealing a substantial reduction in three-year target lesion failure, primarily driven by hard endpoints such as cardiac death and target vessel myocardial infarction [7]. Additionally, a recent systematic review and meta-analysis comparing IVUS- and angio-guided PCI in AMI patients demonstrated a significant association between IVUS guidance and reduced risks of all-cause mortality, major adverse cardiovascular events (MACE), and target vessel revascularization [8]. The most recent revascularization guidelines recommend IVUS in left main artery lesions, where coronary angiography may face limitations due to vessel overlap or foreshortening [9]. However, the role of IVUS in STEMI remains a subject of ongoing research, and its definitive place in this setting is still not well established.

In the acute setting of STEMI, where time is critical, the primary goal is achieving rapid reperfusion through primary PCI. Consequently, conventional practice heavily relies on coronary angiography alone for decision-making during these crucial moments. It offers vital information about the location and severity of coronary artery occlusion, guiding interventionalists in determining the optimal site for stent placement. In this case, the patient presented within the appropriate door-to-balloon time frame for primary PCI. Initial angiographic views revealed thrombotic occlusion of the left main coronary artery (LMCA) extending into the proximal LAD artery. The treatment options for LMCA occlusion vary, including coronary artery bypass grafting (CABG), stent implantation, intracoronary thrombolysis, anticoagulation with heparin or glycoprotein IIb/IIIa inhibitors, and thrombus aspiration [10]. An IVUS can provide a more comprehensive perspective and offer detailed vessel wall characteristics [11]. Given its capacity to elucidate the mechanism of stent failure with cross-sectional images, IVUS is currently considered reasonable [9]. Therefore, it may be judicious to consider IVUS when the etiology of the STEMI is uncertain, as it can help determine the appropriate management strategy.

In this patient with PV and non-compliance with antiplatelet therapy, a hypercoagulable state leading to coronary thrombosis was strongly suspected as the cause of the STEMI. Initially, the management strategy aimed toward mechanical aspiration alongside parenteral antiplatelet and anticoagulation therapy, which appeared reasonable and is supported by some case reports [12-14]. However, a crucial aspect of his medical history is his frequent marijuana use. Studies have demonstrated that marijuana can adversely affect cardiovascular health by interacting with CB1 receptors, leading to the generation of reactive oxygen species and endothelial injury, both of which are implicated in atherosclerosis development [15]. In the absence of other substantial risk factors, it becomes plausible that the patient's marijuana use played a substantial role in the early onset of coronary artery disease. An IVUS further confirmed this suspicion by revealing significant plaque buildup in the ostial LAD, ultimately confirming plaque rupture as the underlying cause of the patient's STEMI and providing support for the decision to proceed with stent implantation.

## Conclusions

While IVUS is not routinely employed in AMI, emerging scientific literature indicates its potential to improve patient outcomes in specific clinical situations. The use of IVUS in this patient with a STEMI of uncertain origin confirmed plaque rupture, providing vital clarity that justified stent implantation tailored to the patient's condition, potentially leading to enhanced outcomes. As we further investigate the advantages of this technology, it becomes increasingly clear that IVUS can play a valuable role in selected AMI cases, enabling more individualized and precise treatment approaches.
